# Surviving Coral Bleaching Events: *Porites* Growth Anomalies on the Great Barrier Reef

**DOI:** 10.1371/journal.pone.0088720

**Published:** 2014-02-19

**Authors:** Neal E. Cantin, Janice M. Lough

**Affiliations:** Australian Institute of Marine Science, Townsville, Queensland, Australia; University of New South Wales, Australia

## Abstract

Mass coral bleaching affected large parts of the Great Barrier Reef (GBR) in 1998 and 2002. In this study, we assessed if signatures of these major thermal stress events were recorded in the growth characteristics of massive *Porites* colonies. In 2005 a suite of short (<50 cm) cores were collected from apparently healthy, surviving *Porites* colonies, from reefs in the central GBR (18–19°S) that have documented observations of widespread bleaching. Sites included inshore (Nelly Bay, Pandora Reef), annually affected by freshwater flood events, midshelf (Rib Reef), only occasionally affected by freshwater floods and offshore (Myrmidon Reef) locations primarily exposed to open ocean conditions. Annual growth characteristics (extension, density and calcification) were measured in 144 cores from 79 coral colonies and analysed over the common 24-year period, 1980–2003. Visual examination of the annual density bands revealed growth hiatuses associated with the bleaching years in the form of abrupt decreases in annual linear extension rates, high density stress bands and partial mortality. The 1998 mass-bleaching event reduced *Porites* calcification by 13 and 18% on the two inshore locations for 4 years, followed by recovery to baseline calcification rates in 2002. Evidence of partial mortality was apparent in 10% of the offshore colonies in 2002; however no significant effects of the bleaching events were evident in the calcification rates at the mid shelf and offshore sites. These results highlight the spatial variation of mass bleaching events and that all reef locations within the GBR were not equally stressed by the 1998 and 2002 mass bleaching events, as some models tend to suggest, which enabled recovery of calcification on the GBR within 4 years. The dynamics in annual calcification rates and recovery displayed here should be used to improve model outputs that project how coral calcification will respond to ongoing warming of the tropical oceans.

## Introduction

Mass coral bleaching events due to thermal stress have been occurring more frequently since the 1980s (see review by [1]). This ecological response is now clearly linked with warming of the tropical oceans [2] associated with human-induced climate change [3]. The occurrence, intensity and responses to coral bleaching conditions show considerable variability. Different coral taxa show different susceptibility to thermal stress, with branching corals often being more sensitive than massive species [4]–[8]. Large-scale field surveys also demonstrate spatial variability in the occurrence and intensity of bleaching within and between coral reefs and across coral reef ecosystems [9]–[11]. Calm and clear conditions can significantly promote coral bleaching events as water motion slows and ocean temperatures rapidly increase. Field observations have found, for example, reduced bleaching incidence in regions of strong water motion due to tides, upwelling or high wave energy which prevents the water column from heating as much as other parts of the reef [12]–[14]. Periods of cloudy weather, followed by increased winds can also significantly dissipate warm pools of water and radiative stress conditions that are conducive to bleaching conditions [15]. The degree of sensitivity to thermal stress may also vary with the predominant type of *Symbiodinium* associated with each coral colony [16]–[18]. On a reef affected by thermal stress, some corals may not bleach, some may partially bleach, some may totally bleach and, once the stress is removed, corals may partially or fully recover and some may die. There is, therefore, also considerable variability in recovery from a bleaching disturbance [1] with absence of other local environmental stressors playing an important role in determining how well particular coral reefs may recover [19]–[21]. Such variable responses to thermal stress within a reef have both short and long-term consequences for the makeup of the hard coral community and associated reef organisms [22]–[23].

The Great Barrier Reef (GBR), Australia experienced high thermal stress and observations of significant coral bleaching in the summers of 1998 and 2002, with 42% and 54%, respectively, of the reef showing some degree of bleaching [10]. The purpose of the present study is to determine what, if any, signatures of these 1998 and 2002 mass coral bleaching events are evident in the annual growth records contained in coral cores from apparently healthy massive *Porites* colonies from four reef sites in the central GBR that survived the worst bleaching events ever recorded in the GBR [10].

Certain massive corals contain annual density banding [24] that allows recovery of continuous (often over several centuries), dated growth parameters and a wealth of proxy climate and environmental records that predate observations on coral reefs (see review, [25]). Although recent use of these natural coral reef archives has focussed on measuring geochemical tracers as environmental and climatic proxies, the value of the long coral growth histories has recently resurfaced in an era of rapid environmental change in many coral reef ecosystems (see review, [26]). Surviving, apparently healthy massive coral colonies provide an opportunity to retrospectively assess the severity of historical stress events from the calcification response recorded within the annual density bands of the coral skeleton. A common global theme to most of the results from this recent resurgence of calcification histories has shown consistent declines in coral growth and calcification since the late 1990's. Based on continuous annual records in multiple *Porites* corals, Cooper et al. (2008) [27] found evidence for a recent decline in coral growth in the northern GBR that was subsequently found throughout the GBR [28]–[29] from the 1990s to early 2000s. A 30% decrease in linear extension rate since 1998 has also been detected in *Diploastrea* corals in the Red Sea [30]. A region-wide 19% decline in *Porites* calcification rates between 1980 and 2010 has recently been reported across six locations of the Thai-Malay Peninsula of southeast Asia [31]. Several studies which re-measured (i.e. discontinuous) coral growth rates have also found slower growth rates: Bak et al. (2009) [32] reported a ∼10% decrease in linear extension of *Acropora*, Curacao between the early 1970s and early 2000s; Tanzil et al. (2009) [33] found ∼20–24% lower calcification and extension rates for *Porites*, in southern Thailand between the mid 1980s and mid 2000s; and Manzello (2010) [34] found that *Pocillopora damicornis* in the eastern Pacific showed ∼30% reduction in linear extension rates between the mid 1970s and mid 2000s. Conversely, a recent study has shown a 10% increase in coral calcification in the central mid-shelf and offshore GBR up to 2008 but an ongoing decline on inshore reefs [35]. *Porites* calcification rates from 1900–2010 within reefs in the southeast Indian Ocean off the coast of Western Australia have also shown 6–23% increases in recent decades [36]. The causes of the observed declines in calcification have been attributed, in part, to increased frequency of thermal stress events that expose corals to conditions that exceed their upper thermal limit [30], [37].

As well as providing continuous retrospective time series of coral growth Knutson et al. (1972) [24] noted the potential for annual density bands to record periods of environmental stress and Macintyre and Smith (1974) [38] suggested that visible growth hiatuses in X-rays could be applied to establishing the frequency of “catastrophic” mortality events on coral reefs. Responses to environmental stress were recorded as abnormal density bands, termed “stress bands”, in multiple *Montastrea annularis* (taxonomy has recently re-classified the *M. annularis* complex as the *Orbicella annularis* complex [39]) colonies examined in Florida [40]. The 1987–1988 mass coral bleaching event did not appear to affect growth rates in unbleached colonies of the *O. annularis* complex in Jamaica but resulted in cessation of growth for ∼6 months in those colonies that partially bleached and survived [41]. Examining the impact of the same thermal stress event on *O. annularis* growth in Florida, Leder et al. (1991) [42] found no evidence of sub-annual stress bands, they did find in some colonies a marked decrease in linear extension rate and loss of part of an annual band but other colonies showed no clear response. Mendes and Woodley (2002) [43] measured several characteristics (e.g. growth, reproduction, tissue depth) in 15 *O. annularis* colonies in Jamaica that lived through the 1995 bleaching event. Seven of the 15 colonies did not visually bleach while the remainder sustained mild paling to severe bleaching categorized as complete loss of visible colour. They found for all colonies (whether visually bleached or not), that the 1995 annual high density band tended to be “more prominent” and sometimes wider than usual and that the polyp tissue depth was significantly negatively correlated with the duration of the bleaching symptoms. They also found that linear extension rates were not significantly different between colonies that did or did not visually bleach before and in the years following the 1995 disturbance. However they did observe that during the event severely bleached colonies showed a 40% reduction in annual skeletal extension rates. This result indicates that the 1995 bleaching event did not have a prolonged impact on skeletal extension rates, however reproduction was inhibited for 2 years following the event. Examining *Porites* growth rates associated with the 1991 thermal stress event at Phuket, Thailand, Tudhope et al. (1993) [44] noted considerable variability in the occurrence of bleaching in *Porites* colonies from the same environment, with ∼50% of colonies visually bleaching. They also found (from alizarin staining) that unbleached corals grew faster than bleached corals during the event, though the latter were still calcifying. They also found no evidence that corals that bleached had significantly different growth characteristics (extension, density or calcification) compared with those colonies that did not bleach for the two years prior to the bleaching event. They concluded that skeletogenesis and the tendency to bleach are controlled by different physiological parameters which, subsequent studies have shown, to be in part related to the diversity of thermal tolerance of different *Symbiodinium* types within the coral host (e.g. [16], [45]–[47]). Previous coral bleaching events throughout a wide range of tropical coral reef environments have been recorded in the annual banding patterns of massive coral skeletons in the form of more prominent high density stress bands and reductions in annual skeletal extension rates.

In a comprehensive study, Carilli et al. (2009) [21] measured linear extension, density and calcification rates in 92 *Montastrea faveolata* coral cores from four sites on the Mesoamerican Reef that varied according to exposure to local anthropogenic stresses. They found evidence that, as a consequence of the 1998 coral bleaching event, coral growth at sites with high local stress suffered suppressed growth for at least 8 years after the event, whereas this “recovery” time was only 2–3 years at sites with low anthropogenic stress. They also noted that nearly all sampled cores showed on X-rays a prominent stress band in 1998. Their observations from field studies that exposure to chronic local stressors (e.g. poor water quality) reduces the ability of corals to recover from episodic stress events (e.g. thermal stress causing coral bleaching) supports the more theoretically-based conclusions that improving local water quality can enhance the resilience of coral reefs to thermal stress events (e.g. Wooldridge, 2009, Wooldridge and Done 2009). Extending coral growth records over the past 75–150 years, Carilli et al. (2010) [48] and Cantin et al. (2010) [30] have concluded that the prolonged impacts on coral growth for 8–10 years following the 1998 mass coral bleaching events in both the Caribbean and Red Sea were unprecedented over the past century, despite periods of higher SST in the past.

The purpose of the present study was to assess what, if any, signatures of the 1998 and 2002 GBR mass coral bleaching events were recorded in the 20–30 year growth characteristics retained in a suite of short *Porites* coral cores collected from surviving colonies from four reef sites in the central GBR. These range from inshore (Nelly Bay, Pandora Reef) sites annually affected by freshwater flood events (and whatever terrigenous material these flood waters may contain), to a midshelf site (Rib Reef) only occasionally affected by freshwater inputs during major flood events to an offshore site (Myrmidon Reef) primarily exposed to open ocean conditions.

## Materials and Methods

### Coral samples and growth variables

**Figure 1:**
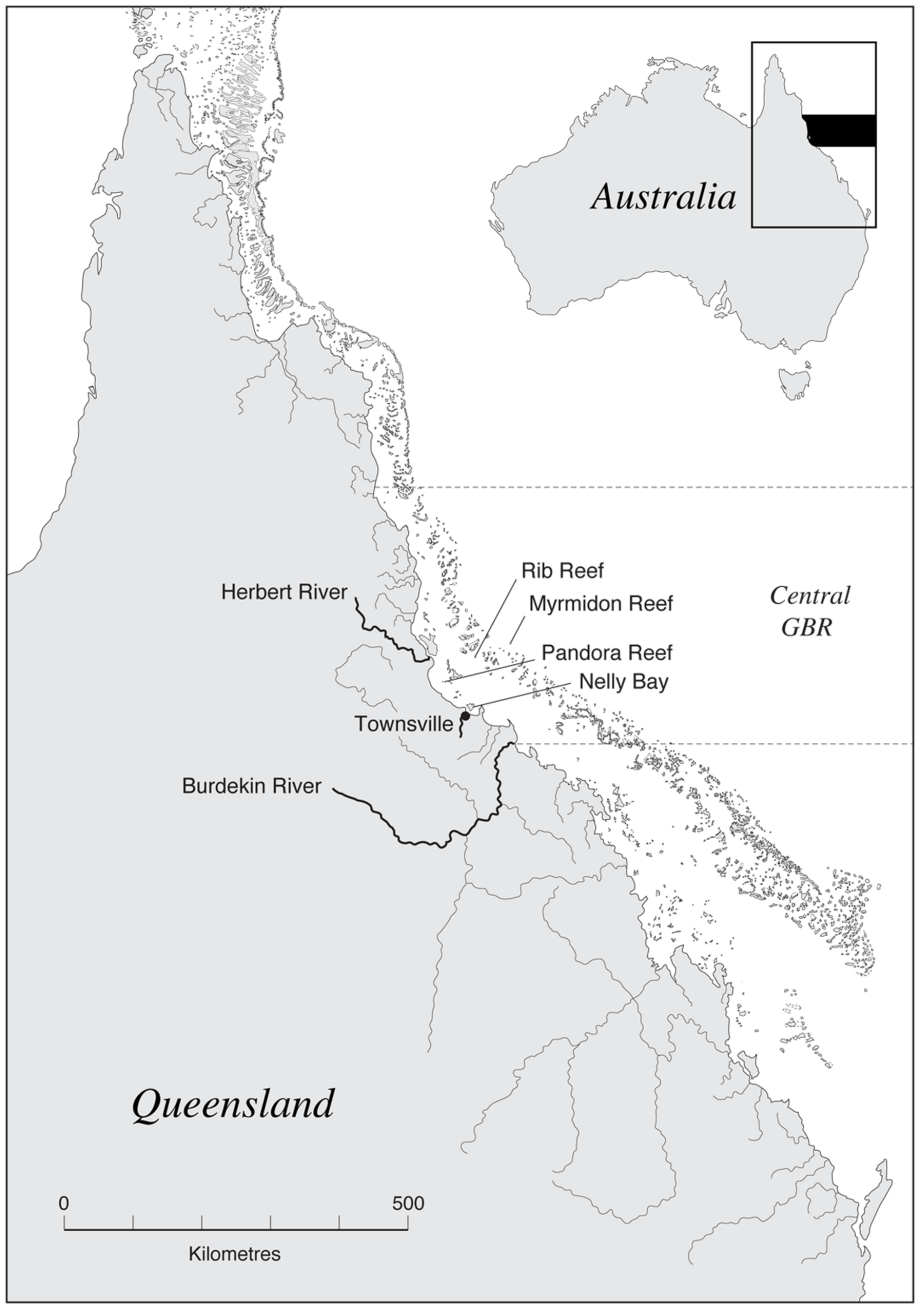
Location of four reef sites in central Great Barrier Reef.

Between May 2004 and October 2005, short (<50 cm) coral cores were collected from 90 *Porites* colonies from four reef environments of the central GBR ∼18–19°S. Two cores were collected from each coral colony (making a total of 186 cores) to increase the likelihood of observing banding patterns along the maximum growth axis. Colonies were from shallow-water environments (∼4–7 m depth) with average colony heights ∼1–2 m (Table 1). Collections from *Porites* colonies within each reef included multiple sites across the entire perimeter of the leeward side of the reef for each location. The reef sites covered a cross-shelf gradient from offshore Myrmidon Reef (∼110 km from land), through mid-shelf Rib Reef (∼56 km from land) to inshore Pandora Reef (∼16 km from land) and Nelly Bay on Magnetic Island (<1 km from land) (**Figure 1**).

**Table 1:** Collection details of coral cores including reef location, depth (m), colony height (m), total number of cores collected and included in growth analyses.

Reef	Latitude	Longitude	Average water depth (m)	Average depth to colony surface (m)	Average colony height (m)	Total coral colonies (#)	Total cores (#)	Cores analysed for growth (#)
Nelly Bay	19.2°S	146.9°E	4.58±1.28	3.60±1.22	1.05±0.51	24	48	25
Pandora	18.8°S	146.4°E	4.12±1.31	2.72±1.16	1.43±0.69	21	42	39
Rib	18.5°S	146.9°E	5.72±1.04	3.90±1.10	1.74±0.76	22	44	42
Myrmidon	18.3°S	147.4°E	6.32±1.38	4.33±1.34	1.73±0.89	23	46	38

**Figure 2:**
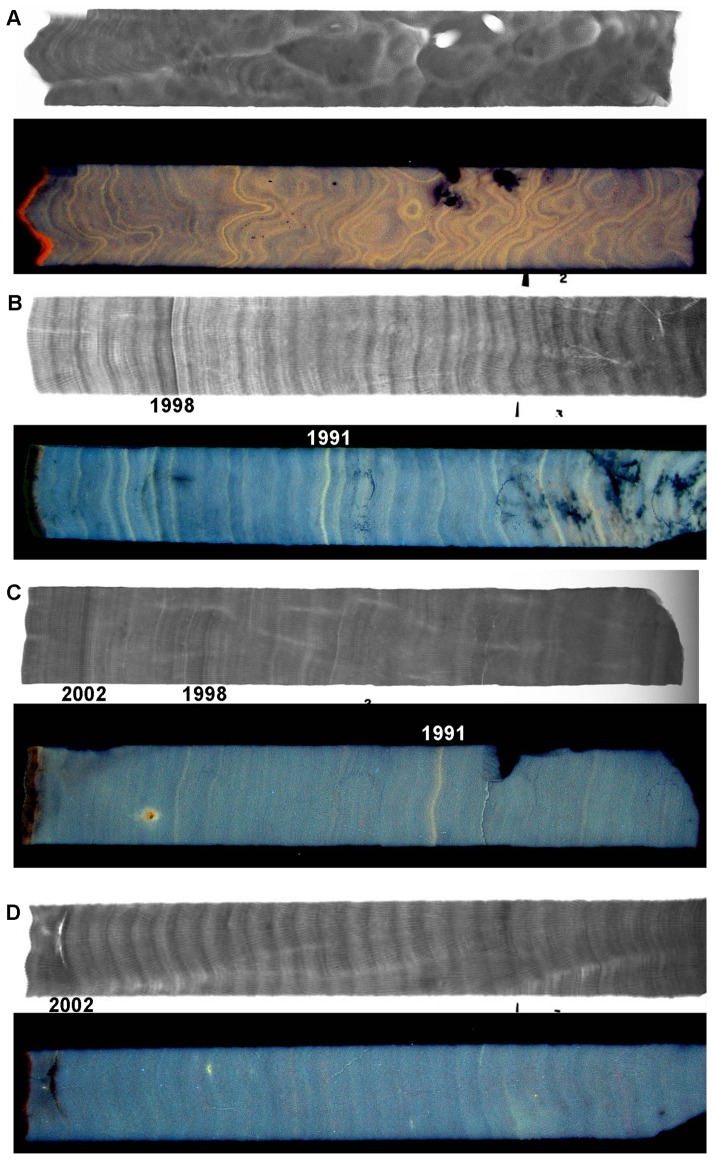
Example positive X-ray prints of short core slices showing annual density banding and photographs under UV light showing luminescent lines and bands. (A) Nelly Bay (Nel 21A_S2) illustrating convoluted growth typical of this inshore site and annual luminescent lines (*off axis core such as this not included in calcification analysis) (B) Pandora (Pan 41A_S3) illustrating 1998 growth hiatus and annual luminescent lines, (C) Rib (Rib 23B_S2) illustrating growth hiatuses in 1998 and 2002 and a bright luminescent line in 1991, and (D) Myrmidon (Myr 35B_S3) illustrating regular density banding typical of this offshore site with faint luminescent bands (minimal flood exposure) and growth hiatus from partial mortality in 2002. X-ray positive: high density  =  dark greys, low density  =  white.

Coral slices were prepared using standard techniques [49]–[50]: cores were air dried, mounted on aluminium trays and three, 6–7 mm thick slices, removed. Each slice was then X-rayed and converted to a positive print (**Figure 2**). Density was measured using gamma densitometry along the slice from each core that showed the clearest presentation of annual density bands [51]. The intensity of luminescence was also measured along each track [52].

Based on the annual high density band forming in summer and the annual low density band in winter, successive density peaks were dated from the date of collection of each core. For inshore and midshelf reefs, additional chronological control was provided by the regular (inshore) and occasional (midshelf) occurrence of bright luminescent lines associated with high river flow events (**Figure 2**; [53]–[55]).

Once dated, the following variables were extracted for each coral slice [56]: 1) annual linear extension (cm.yr^−1^), measured as the linear distance between adjacent low density minima, 2) annual average skeletal density (g.cm^−3^), measured as the average density between low density minima, 3) annual calcification rate (g.cm^−2^.yr^−1^), calculated as the product of annual extension and annual density, 4) annual luminescence range, measured as the difference between peak luminescence of the current summer and that of the preceding winter [57], and 5) average depth of the living tissue layer at the outer surface of the slice [58].

Not all coral slices clearly presented annual density bands suitable for extracting annual growth parameters over several years [59]–[60]. As a consequence, annually dated growth variables were obtained for between 52% (Nelly Bay) and 95% (Rib Reef) of the total number of cores collected from each reef (Table 1). Analyses were undertaken over the 24-year period, 1980–2003 common to all cores with measurable growth variables.

**Figure 3:**
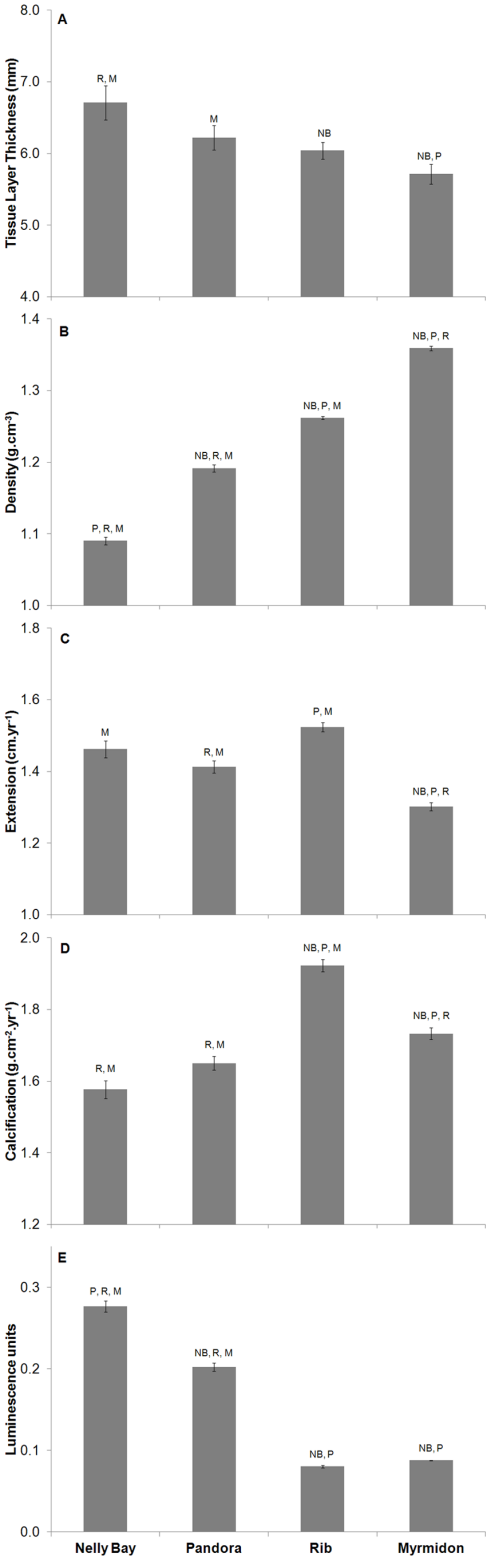
Growth characteristics averaged by reef site (± se), 1980–2003. (A) tissue layer thickness, (B) skeletal density, (C) linear extension rate, (D) calcification rate, and (E) annual luminescence range. (reef sites arranged from inshore to offshore according the water quality characteristics (Table 2). Statistically distinguishable sites are identified by the letters above each bar (NB-Nelly Bay, P-Pandora Reef, R-Rib Reef and M-Myrmidon Reef) at α = 0.05.

Annual linear extension, density, calcification and luminescence range for each available year and dated slice were standardized (to allow for differences in absolute values between corals) with the respective 1980–1997 mean. These anomalies were then averaged by reef for each year and expressed as a percent change from the pre-stress 1980–1997 baseline value. Annual *Porites* growth data (linear extension, density and calcification rates) can be obtained from e-atlas (http://e-atlas.org.au/content/coral-calcification). Reef location comparisons of average colony growth characteristics (tissue thickness layer (TTL), density, extension, calcification and luminescence units; **Figure 3**) were conducted using a One-Way ANOVA (α = 0.05) and a Tukey-Kramer post-hoc test to distinguish significant differences for each location.

### Visual and quantitative assessment of growth events in Porites cores

The X-ray positive of slices from each coral core were examined for evidence of a growth hiatus in 1998 and 2002 (**Figure 2**). A growth hiatus was defined as either visible partial mortality within the core (**Figure 2D**; Myr35B_S3) or obvious high density stress bands, followed by suppressed annual extension (**Figure 2B**; Pan41A_S3). The percentage of the total number of cores at each site which exhibited a visual growth anomaly in 1998 and 2002 was calculated for each year individually. The percentage of colonies that exhibited repetitive visual growth anomalies for both events was also quantified. Annual calcification rates within each reef location following the 1998 warming event for each core were also compared to baseline pre-stress mean calcification rates (1980–1997) to quantify the percentage of cores with significant negative calcification anomalies (calcification rate <95% confidence limits; [61]) resulting from each bleaching event in 1998 and 2002. Linear regression analysis was conducted for annual extension, density and calcification rates for each site. Assumptions for linear regression analysis were tested using residual plot analyses.

### Environmental variables

Water characteristics (chlorophyll concentration and secchi extinction depth) were obtained for each site (http://e-atlas.org.au/; see [62]). Annual, October –September, total Burdekin River flow was obtained from the Queensland Department of Environment and Resource Management (http://www.derm.qld.gov.au/water/).

To assess the magnitude of thermal stress that each site may have experienced during the 1998 and 2002 bleaching events, SST data were obtained from 1) average monthly 1-degree latitude by longitude boxes between 18.5–19.5°S, 146.5–147.5°E, encompassing the four reef sites (SSTs for the 3 boxes were highly and significantly correlated, r≥0.99), 1980–2003, HadISST [63], and 2) weekly 4 km NOAA satellite fields, 1985–2003 [64]. Annual maximum SST anomalies were calculated from the monthly SST data for the period 1980–2003. From the weekly high-resolution SSTs, the accumulated degree heating weeks (relative to the average weekly maximum, 1985–2003 base period) was calculated for each site for 1998 and 2002.

## Results

### Environmental conditions

The four reef sites encompass an inshore to offshore gradient in water quality characteristics as measured by average chlorophyll concentration and secchi disk extinction depth (Table 2). Clearest waters with low chlorophyll concentration are found at the shelf edge Myrmidon Reef with most turbid waters with high chlorophyll concentration in Nelly Bay. Values at Pandora Reef (inshore but further from land) are similar to Nelly Bay and conditions at mid-shelf Rib Reef are most similar to Myrmidon Reef.

**Table 2:** Summary of water characteristics at reef sites (De'ath & Fabricius 2010; e-atlas.org.au).

Reef	Chlorophyll (mg L^−1^)	Secchi depth (m)
Nelly Bay	0.69	5.4
Pandora	0.48	5.8
Rib	0.23	19.9
Myrmidon*	<0.21	>26.2
GBR range	0.14–1.67	2.8–27.9

* Myrmidon not in e-atlas so estimated from nearest shelf-edge reef (Dip Reef: 17 km SE of Myrmidon reef).

**Figure 4:**
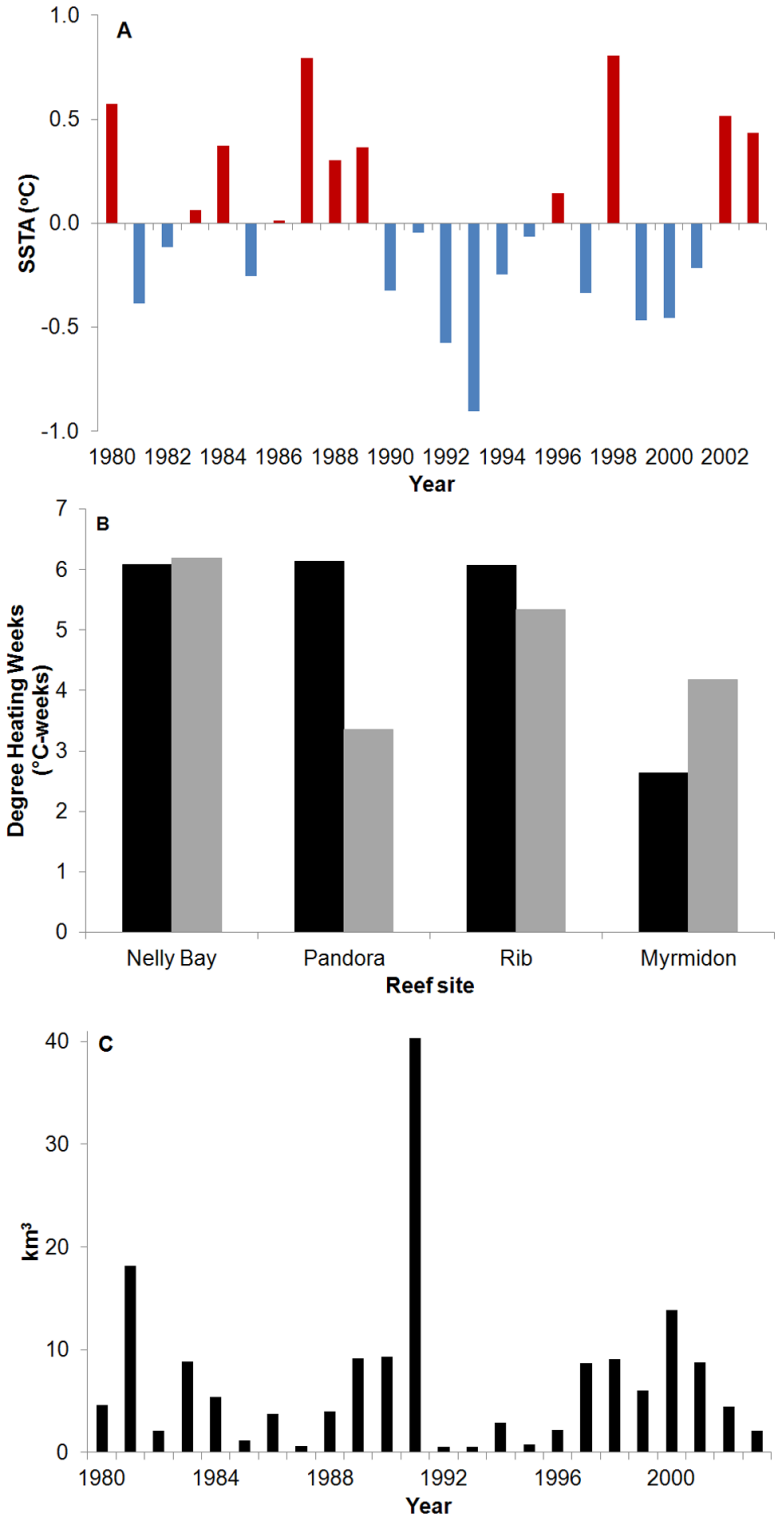
Historical thermal stress and flood plume intensity for the Central Great Barrier Reef, 1980-2003. (A) Annual maximum SST anomalies, 1980-2003 (HadISST1, Rayner et al. 2003), (B) Accumulated 4km Degree Heating Weeks at four reef sites (NOAA data) for 1998 (black) and 2002 (grey), and (C) annual (October-September) Burdekin River flow, 1980-2003 (Queensland Department of Environment and Resource Management www.derm.qld.gov.au/water).

The warmest annual maximum SST anomaly, over the period 1980–2003, occurred in 1998 and 2002 was the fourth warmest year after 1998, 1987 and 1980 (**Figure 4A**). The level of thermal stress for these years, as estimated from the NOAA 4 km Degree Heating Week (DHW) product, was similar and high at levels that predict significant coral bleaching (>4°C-weeks; [65]) at Nelly Bay and Rib Reef, about twice as high in 1998 than 2002 at Pandora Reef and lowest at Myrmidon Reef which appeared to experience greater thermal stress in 2002 compared to 1998 (**Figure 4B**).

**Figure 5:**
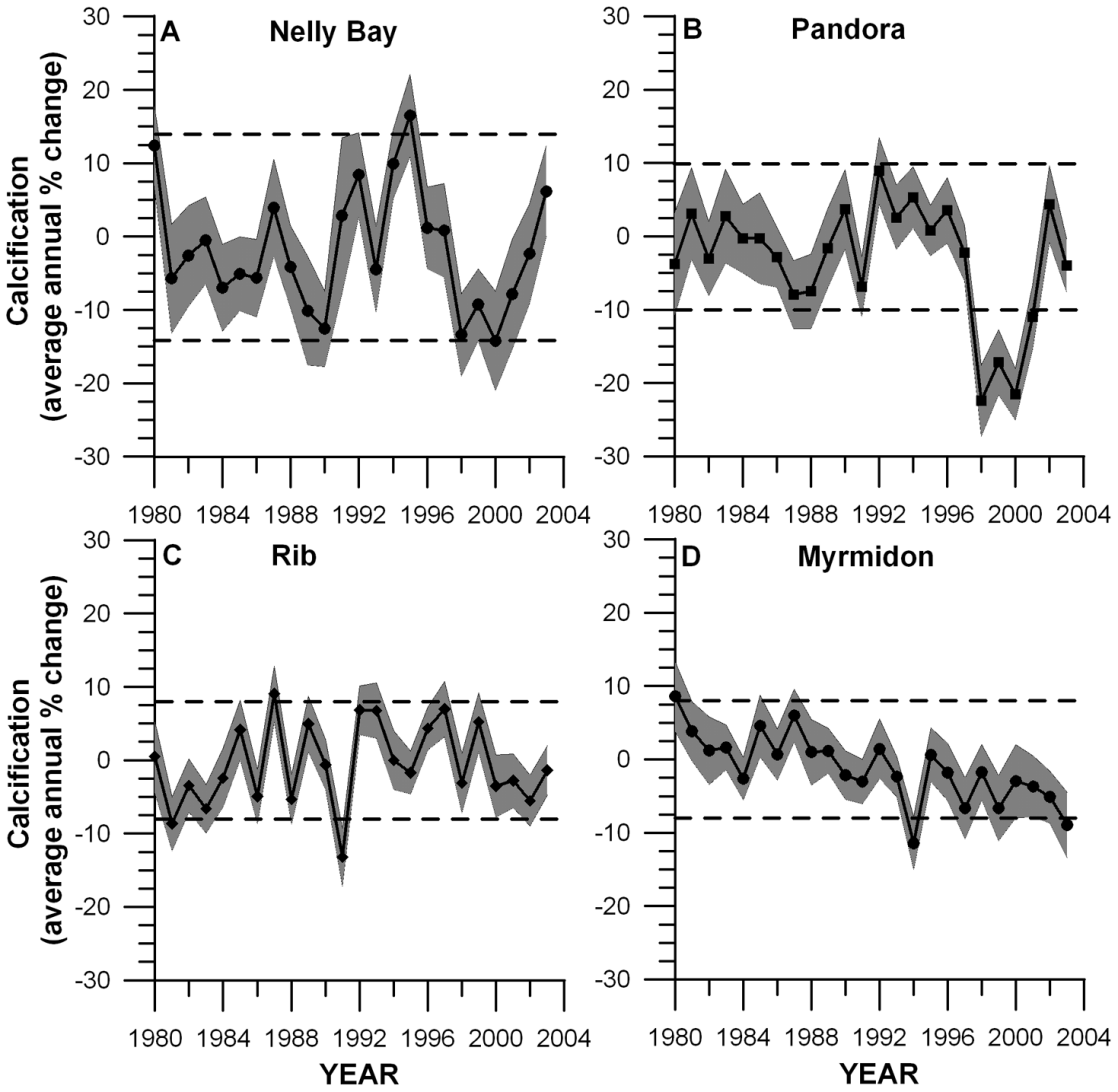
Average annual standardized *Porites* calcification anomaly time series from the Central Great Barrier Reef (1980–2003), as a percent difference from mean baseline calcification rates (1980–97) prior to the 1998 mass bleaching event (± SE). A) Nelly Bay (1980–97 baseline calcification  = 1.61±0.12 g cm^−2^ yr^−1^; 95% CI: ±14.3%), B) Pandora Reef (1980–97 baseline calcification  = 1.69±0.08 g cm^−2^ yr^−1^; 95% CI: ±10.1%), C) Rib Reef (1980–97 baseline calcification  = 1.69±0.08 g cm^−2^ yr^−1^; 95% CI: ±8.5%) and D) Myrmidon Reef (1980–97 baseline calcification  = 1.92±0.08 g cm^−2^ yr^−1^; 95% CI: ±8.6%). Shading represents ±1 standard error for each annual mean calcification anomaly. Dashed lines represent ±95% confidence intervals (CI) around the baseline calcification mean (1980–97).

An additional source of stress, at least in the inshore and occasionally mid-shelf sites, is summer freshwater flood plumes. Annual freshwater flows from the Burdekin River, which regularly affects Pandora Reef and Nelly Bay, shows that within the period of study, 1980–2003, the most exceptional event was the vast flood plume that occurred in 1991 (**Figure 4C**). The total flow in this year was ∼480% of the median flow of the Burdekin River since gauged records started in 1922. Transport of this flood plume to the mid-shelf Rib reef location was recorded within the coral cores, as one of the very few visible luminescent lines during this 24 year time period (**Figure 2C**) and a negative calcification anomaly was observed at Rib Reef in 1991 (**Figure 5C**).

### Average characteristics by reef

Tissue layer thickness was highly variable but, on average, showed a significant gradient from high to low from the inshore (Nelly Bay and Pandora Reef) to offshore (Myrmidon Reef) site (**Figure 3A**, One-Way ANOVA: F_3, 140_ = 5.61, p = 0.001) that reflected the gradient in water quality characteristics (Table 2). Skeletal density showed the opposite gradient, increasing from inshore to offshore sites (**Figure 3B**, One-Way ANOVA: F_3, 3165_ = 703.04, p<0.001). Extension and calcification rates were both variable across the environmental gradient but tended to be highest at the mid-shelf, Rib Reef, site (**Figures 3C**, One Way ANOVA: F_3, 3165_ = 34.27, p<0.001 **and 3D**, One Way ANOVA: F_3, 3165_ = 59.26, p<0.001). As noted in previous studies [66], skeletal density and extension rate were significantly inversely related (r = −0.39, n = 144) and linear extension rate was the primary source of variability in calcification rate (r = 0.83, n = 144) with no significant correlation with skeletal density (r = 0.12, n = 144, based on core averages). The luminescence range (a measure of freshwater influence) was greatest at Nelly Bay and Pandora Reef – regularly affected by annual flood events from the Burdekin River with similar low values at the midshelf and offshore sites (**Figure 3E**, One Way ANOVA: F_3, 2724_ = 530.34, p<0.001).

### Visual assessment of 1998 and 2002 growth anomalies

**Figure 6:**
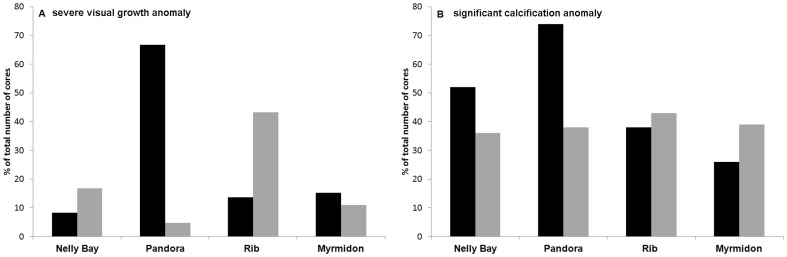
Growth anomalies recorded in *Porites* calcification histories, percentage of coral cores at each reef site showing. (A) visual growth anomaly (partial mortality, high density stress band, visual reduced annual extension) and (B) significant negative calcification anomaly (significant reduction in annual calcification compared to the 1980–97 mean baseline; 95% confidence intervals dashed lines Figure 6). 1998 (black) and 2002 (grey).

The occurrence of growth anomalies identified in the X-ray positive prints varied considerably between reef sites and between 1998 and 2002 (**Figure 6A**). Nearly 70% of 21 coral colonies at Pandora Reef showed a visual growth hiatus in 1998 but only 5% in 2002. Conversely only 14% of the cores from Rib Reef showed a growth anomaly in 1998, but 40% of the cores contained a growth anomaly in 2002. Nelly Bay also showed a greater number of visual anomalies in 2002 compared with 1998 but values were relatively low (17% and 8%, respectively). Cores from the offshore Myrmidon Reef showed similar and relatively low frequency of visual growth anomalies during both of the years. At Rib Reef, which had the highest level of bleaching during the second thermal anomaly in 2002, a very low percentage of colonies that bleached in 1998 showed signs of adaptation with 2 of the 3 colonies that bleached in 1998 showing repetitive growth anomalies. While a low percentage of the Nelly Bay colonies exhibited visual growth anomalies, ∼50 and 30% of the colonies exhibited significantly lower calcification rates in 1998 and 2002 respectively (**Figure 6B**). A similar pattern was apparent for colonies from Pandora reef in 2002, with <5% of colonies exhibiting visual growth anomalies, however 35% colonies suffered reduced calcification rates in 2002 (**Figure 6B**).

### Time series of calcification anomalies

**Figure 7:**
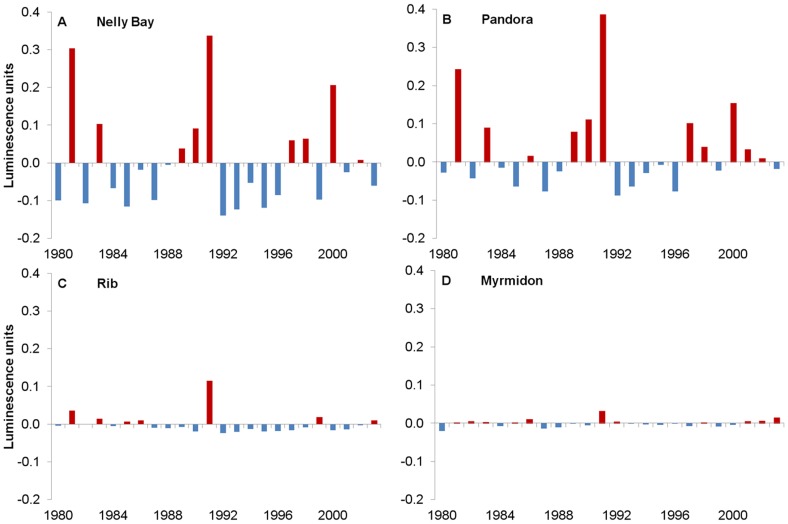
Average annual anomalies of luminescence range, 1980–1983 from coral slices. Significant positive anomaly in 1991 reflects major flood plumes transported to reefs in the inshore and mid-shelf reefs. **Inshore sites**: (A) Nelly Bay, (B) Pandora Reef, **Mid-shelf site:** (C) Rib Reef, **Offshore site:** (D) Myrmidon Reef.

Average reef standardized calcification anomalies (% change compared to the baseline 1980–1997 mean calcification rate) over the period 1980–2003 showed a number of features which varied with reef. Nelly Bay appeared to have experienced relatively high and similar magnitude thermal stress in 1998 and 2002 (**Figure 4B**), but a relatively low percentage of cores with visual growth anomalies (**Figure 6A**). Annual linear extension and calcification (**Figure 5A**), however, were below average from 1998 to 2002. The annual luminescence range (**Figure 7A**) was significantly correlated with Burdekin River flow (**Figure 4C**; r = 0.83, p<0.05) reflecting the freshwater influence at this nearshore site.

A similar suppression of linear extension and calcification rates from 1998 to 2001 was also evident at Pandora Reef (**Figure 5B**), the reef which had relatively high thermal stress in 1998 and less so in 2002 (**Figure 4B**) but which had nearly 70% of cores exhibiting a growth anomaly in 1998 (**Figure 6**). The strong annual freshwater influence at this site is corroborated by the significant correlation between the luminescence range and Burdekin River flow (r = 0.94, p<0.05).

Average growth anomalies at Rib Reef (**Figure 5C**) were less marked compared to the two inshore sites despite similar levels of high thermal stress in 1998 and 2002 (**Figure 4B**) and ∼40% of cores showing a visual growth anomaly in 2002 (**Figure 6A**). The largest negative anomaly evident in linear extension, density (-2%) and calcification rate (-13%) occurred in 1991 which is likely a result of low salinity stress associated with the large flood of that year, when freshwater extended as far as this mid-shelf reef, as indicated by the large 1991 anomaly of the luminescent range (**Figure 7C**) and the clear luminescent line visible within the core (**Figure 2C**).

Thermal stress at Myrmidon Reef was relatively low compared to the other three sites in both 1998 and 2002 (**Figure 4B**), as was the appearance of visual growth anomalies in these two years (**Figure 6**). The calcification anomalies (**Figure 5D**) were relatively small, although density (R^2^ = 0.66, p<0.05), linear extension (R^2^ = 0.27, p<0.05) and calcification rates (R^2^ = 0.53, p<0.05) all show a significant 11% linear decrease over the period 1980–2003.

## Discussion

Historical calcification records from the annual density banding patterns within the skeletons of surviving *Porites* coral colonies have been used to assess the impact of recent bleaching events in the GBR, across an inshore-offshore gradient. Analysis of 144 cores, from 79 *Porites* colonies, across 4 different reef locations reveals that the 1998 thermal anomalies impacted calcification on the inshore locations more than the 2002 event. Pandora reef was the most severely impacted location by the 1998 SST anomaly, which corresponds with visual growth anomalies in the form of high density stress bands and reduced annual linear extension and calcification in ∼70% of the colonies. Following the 1998 bleaching event an 18% and 13% reduction in *Porites* calcification was observed at Pandora Reef and Nelly Bay, respectively. Recovery of *Porites* calcification to pre-1998 baseline rates did not occur for 4 years until 2002 at both inshore locations.

Detailed mapping of the 1998 and 2002 bleaching events was conducted on the GBR with aerial and *in situ* surveys describing the spatial distribution of the disturbance events [10], [67]. Extreme levels of bleaching (>60% coral cover bleached) occurred on 28% of the reefs in the central GBR in 1998 [67]. More intense bleaching was observed on inshore reefs during both events, however the extent of bleaching on offshore sites increased from 21% in 1998 to 41% in 2002 [10]. Very high levels of bleaching occurred at the inshore sites, Pandora Reef and Nelly Bay, with 30–60% of coral cover visually bleached in 1998 [67]. Pandora Reef exhibited less than 1% bleaching in 2002, which supports the absence of visual growth anomalies in our *Porites* cores collected from the surviving colonies. Nelly Bay suffered equal bleaching severity in 1998 and 2002 with approximately 30–50% of the coral cover bleached during both events [10], [67]. While these results, particularly the aerial surveys, largely documented the bleaching status of the more sensitive dominant branching coral species (*Acropora* and *Pocillopora*) from the reef crest and upper reef slope (depths <6 m, [10]) that are visible from the air, similar patterns of stress from each event were detected within the calcification histories of surviving massive *Porites* corals from the central GBR. Our calcification records show that temperature stress that caused widespread bleaching in 1998 corresponded with significant growth anomalies in the form of visible high density stress bands, partial mortality, negative extension anomalies and abrupt decreases in *Porites* calcification at the inshore sites Pandora Reef and Nelly Bay. The 2002 event impacted the offshore sites more than 1998, with ∼40% of the cores from Rib Reef displaying visual growth anomalies and evidence of partial mortality in 2002 within 10% of the cores from Myrmidon Reef. Through the combination of detailed documented bleaching surveys at these locations and long-term calcification histories obtained from surviving coral colonies, we can conclude that the observed calcification anomalies within the annual banding patterns of *Porites* skeletons were caused by the associated stress from the mass bleaching events.

Mass bleaching events on coral reefs are important biological disturbances that can reduce colony growth and reproductive output and could lead to reduced reef growth, hard coral cover, species diversity and ecosystem complexity [6], [68]–[69]. Previous ecological studies of mass bleaching events have reported wide differences in bleaching susceptibility that relates to symbiont diversity [16], [70] and growth form, with faster growing branching species more sensitive than slow growing massives [6]. Recently however, an unprecedented reversal of bleaching susceptibility was observed in regions of South East Asia (Singapore), where healthy branching *Acropora sp.* colonies were found adjacent to severely bleached encrusting, foliose and massive corals, including *Porites* [7]. Given these observations, when signs of stress become obvious in the often considered tolerant species such as massive *Porites* colonies, these signatures represent a strong signal of widespread stress within the community.

Experimental and field studies have shown that optimal coral calcification occurs at temperatures that are 1–3°C below seasonal maximums [27], [71] and as conditions exceed typical summertime maximums, even by as little as 0.5°C annual calcification rates decline [30]. Visual growth anomalies (**Figure 2** **&** **6A**) within the annual banding patterns of *Porites* cores represent the impact of severe bleaching and a prolonged period of energetic stress to the individual colony. Severe stress causes high-density stress bands that are visually apparent in the skeleton under X-ray (**Figure 2**) because tissue growth and vertical skeletal extension is impaired for a prolonged period of time while the individual coral colony rebuilds the energetic reserves required for tissue growth [72]. Negative calcification anomalies quantified from the gamma densitometery profile (**Figure 6B**) represent moderate levels of stress that have caused reduced annual growth and calcification rates. Since the same visual growth anomaly was not apparent in some of these cores, it suggests that the energetic recovery period was shorter and as a result normal extension resumed much faster and the appearance of a high density stress band did not occur. A comprehensive study by Thornhill et al. (2011) [73] indicates that a coral colony's tissue biomass and energetic reserve content significantly increases the susceptibility to mortality following periods of severe stress when metabolic reserves are most critical. This study indicates that there is a minimum tissue biomass level, as an indicator of colony health that is required prior to severe bleaching events in order to ensure survival [73]. Likewise, the severity of the calcification response is not only an indication of the severity of the stress event but will also be related to colony condition prior to the event. These two factors will contribute to the rate of recovery and the type of calcification signature left within the banding patterns of the surviving colony.

Comparing the frequency of severe visual growth anomalies and moderate calcification anomalies indicates that ocean temperatures exceeded the optimal range for coral growth in 40–70% of the *Porites* colonies in the central GBR in 1998 and 2002. Conversely, our results possibly indicate that 30% of the *Porites* colonies from Pandora Reef contained the energetic reserves [73] and/or the molecular phenotypic adaptations that contribute to enhanced thermal tolerance levels [16], [74]–[75] that are required to resist the negative effects of the 1998 and subsequently 2002 bleaching events. Calcification histories obtained from the annual density banding patterns of massive corals is a tool that can identify: the frequency of repetitive bleaching responses, adaptation to repeated stress events and coral colonies that are both sensitive and tolerant to major thermal stress events. This technique provides the opportunity to study the physiological attributes of tolerance and will contribute significantly to our understanding of the potential for evolutionary processes to buffer the coral community responses to environmental change and identify critical management strategies to promote resilience for future warming events.

Climate change is widely regarded as one of the greatest threats to coral reef ecosystems, with the biological consequences of warming ocean environments displayed in the increased frequency of coral bleaching in recent decades [1]. Severe thermal stress events capable of causing widespread coral bleaching and mortality are projected to become more frequent over the next 20–50 years in the absence of significant and positive evolutionary enhancement of thermal tolerance [76], which threatens the health and survival of reef-building corals worldwide. The GBR has been considered as the world's least threatened reef system due to its lack of intensive coastal development, strong legal protection and effective resource management as a multi-use marine park since the 1980's [77]–[78]. Despite these advantages, several recent studies have described a general decline in the health and condition of GBR reefs over recent decades, both in terms of living coral cover (∼10–50% estimates of decline, [69], [77]–[79]) and colony calcification (12–21% decline; [27]–[28], [35]). Although there were two major bleaching events (the worst documented on record) recently in 1998 and 2002 on the GBR, there were significant differences in the spatial severity both within each event and between events [10]. Calcification trends on the offshore central GBR location (Myrmidon Reef) displayed a gradual decline of 11% over the entire 24 year period that does not correlate with a distinct disturbance event, other than the few colonies (10%) that recorded partial mortality in 2002. This gradual chronic decline resembles the expected effect on calcification associated with ocean acidification, as coral calcification processes have been shown to become energetically less favourable as CO_2_ is absorbed by the oceans and the aragonite saturation state (Ω_arg_) declines [80]–[81]. However, a much better understanding of carbonate chemistry variability on the offshore GBR is required to extend this interpretation. Coral calcification on the inshore reefs, Pandora Reef and Nelly Bay was impacted severely by the 1998 event but not in 2002. Interestingly, calcification at both sites recovered to pre-1997 baseline levels within 4 years and was increasing at the time of collection in 2004. This observation of calcification recovery for the inshore corals suggests that they are well adapted to the local environmental conditions (e.g. water quality) and are not suffering from local anthropogenic stress [21] that would otherwise impair or prolong their ability to recover from severe disturbance events [37]. There is no evidence of continual decreasing calcification at these inshore and mid-shelf locations in the central GBR, as has been suggested previously for these specific sites [35] and GBR-wide calcification patterns [28]. Our results do, however, clearly show setbacks in coral growth due to stress events. These calcification trends, as an estimate of coral physiological condition, provide optimistic evidence of tolerance within GBR communities to persist through the current rate of severe disturbance events (estimated frequency of 6–11 years, based on shifts in coral cover; [79]) that threatens this World Heritage listed ecosystem.

## Conclusions

Through the combination of documented bleaching records and calcification histories recorded in coral cores from massive *Porites*, we are able to identify calcification signatures from historical disturbance events. Growth signatures associated with coral bleaching that are retained within the coral cores include: 1) an abrupt decrease in annual linear extension and calcification rates, 2) high density stress bands and 3) partial mortality leading to growth hiatuses within individual cores. The second and third signatures become apparent following severe stress. Negative growth anomalies attributed to coral bleaching can be differentiated from stress caused by flooding and low salinity stress through the presence of intense luminescent lines visualized under UV light. Historical growth chronologies from density banding patterns in massive coral skeletons provide a window into the past that enables the quantification of major disturbance events within corals reefs over timescales that exceed current monitoring programs by decades. Now that we have characterised the calcification signatures associated with mass bleaching events we can begin to investigate the historical frequency of major stress events in longer cores that represent centuries of coral reef environmental conditions. When signatures of stress events become obvious in more tolerant species such as massive *Porites* colonies, these signatures represent a strong signal of widespread stress within coral communities. Additionally, calcification records provide an annual assessment of the physiological health and condition of coral colonies before, during and after major stress events, which surveys of coral cover do not. Efforts to combine these common measurements of historical patterns of coral reef ecosystem health will contribute to improve our understanding of how coral reefs are responding to disturbance events from climate change (high temperature induced bleaching) and local land use practices. Since 1980 there have been two major bleaching events on the GBR (1998 & 2002), without an additional major event since 2002. While these events caused widespread bleaching on more than 50% of the GBR, over a short four-year period, our results indicate two major findings: 1) the frequency of severe temperature stress at individual sites has not yet increased as projected, and 2) calcification rates recovered from the effects of severe bleaching within 4 years and have not continued to decline. These two observations and in combination with future efforts to update calcification trends for the GBR that include the last decade following the 2002 event will provide significant advances that will better inform models that project how coral calcification will respond to ongoing warming of the tropical oceans.

## References

[1] Baker AC, Glynn PW, Riegl B (2008) Climate change and coral reef bleaching: An ecological assessment of long-term impacts, recovery trends and future outlook. Estuarine Coastal and Shelf Science 80: 435–471.

[2] Eakin CM, Lough JM, Heron SF (2009) Climate Variability and Change: Monitoring Data and Evidence for Increased Coral Bleaching Stress Coral Bleaching. In: Oppen MJH, Lough JM, editors: Springer Berlin Heidelberg. pp. 41–67.

[3] IPCC (2007) Climate Change 2007: The Physical Science Basis. Cambridge and New York. 996 p.

[4] McClanahan TR, Baird AH, Marshall PA, Toscano MA (2004) Comparing bleaching and mortality responses of hard corals between southern Kenya and the Great Barrier Reef, Australia. Mar Pollut Bull 48: 327–335. 14972585 10.1016/j.marpolbul.2003.08.024

[5] McClanahan TR, Maina J, Moothien-Pillay R, Baker AC (2005) Effects of geography, taxa, water flow and temperature variation on coral bleaching intensity in Mauritius. Marine Ecology Progress Series 298: 131–142.

[6] Marshall PA, Baird AH (2000) Bleaching of corals on the Great Barrier Reef: different susceptibilities among taxa. Coral Reefs 19: 155–163.

[7] Guest JR, Baird AH, Maynard JA, Muttaqin E, Edwards AJ, et al (2012) Contrasting patterns of coral bleaching susceptibility in 2010 suggest an adaptive response to thermal stress. PLoS ONE 7: e33353. 22428027 10.1371/journal.pone.0033353 PMC3302856

[8] Loya Sakai, Yamazato Nakano, Sambali, et al (2001) Coral bleaching: the winners and the losers. Ecology Letters 4: 122–131.

[9] McClanahan TR, Ateweberhan M, Graham NAJ, Wilson SK, Ruiz Sebastian C, et al (2007) Western Indian Ocean coral communities: bleaching responses and susceptibility of extinction. Marine Ecology Progress Series 337: 1–13.

[10] Berkelmans R, De'ath G, Kininmonth S, Skirving W (2004) A comparison of the 1998 and 2002 coral bleaching events on the Great Barrier Reef: spatial correlation, patterns and predictions. Coral Reefs 23: 74–83.

[11] McClanahan TR, Ateweberhan M, Muhando CA, Maina J, Mohammed MS (2007) Effects of climate and seawater temperature variation on coral bleaching and mortality. Ecological Monographs 77: 503–525.

[12] Salm RV, Coles SO (2001) Coral bleaching and marine protected areas. Proceedings of the Workshop on Mitigating Coral Bleaching Impact through MPA Design, Bishop Museum, Honolulu, Hawaii, 29–31 May 2001 Asia Pacific Coastal Marine Program Report # 0103, The Nature Conservancy, Honolulu, Hawaii, USA, 118pp

[13] Skirving W, Guinotte J (2001) The sea surface temperature story on the Great Barrier Reef during the coral bleaching event of 1998. In: Wolanski E, editor. Oceanographic Processes of Coral Reefs. Physical and Biological Links in the Great Barrier Reef. Boca Raton, Florida: CRC Press. pp. 301–313.

[14] Riegl B (2003) Climate change and coral reefs: different effects in two high-latitude areas (Arabian Gulf, South Africa). Coral Reefs 22: 433–446.

[15] Mumby PJ, Chisholm JRM, Edwards AJ, Andrefouet S, Jaubert J (2001) Cloudy weather may have saved Society Island reef corals during the 1998 ENSO event. Marine Ecology Progress Series 222: 209–216.

[16] Berkelmans R, van Oppen MJH (2006) The role of zooxanthellae in the thermal tolerance of corals: a ‘nugget of hope’ for coral reefs in an era of climate change. P Roy Soc Lond B Bio 273: 2305–2312. 10.1098/rspb.2006.3567 PMC1636081 16928632

[17] Maynard JA, Anthony KRN, Marshall P, Masiri I (2008) Major bleaching events can lead to increased thermal tolerance in corals. Marine Biology 155: 172–182.

[18] Oliver TA, Palumbi SR (2009) Distributions of stress-resistant coral symbionts match environmental patterns at local but not regional scales. Marine Ecology Progress Series 222: 209–216.

[19] Sheppard CRC, Harris A, Sheppard ALS (2008) Archipelago-wide coral recovery patterns since 1998 in the Chagos Archipelago, central Indian Ocean. Marine Ecology Progress Series 362: 109–117.

[20] Smith LD, Gilmour JP, Heyward AJ (2008) Resilience of coral communities on an isolated system of reefs following catastrophic mass-bleaching. Coral Reefs 27: 197–205.

[21] Carilli JE, Norris RD, Black BA, Walsh SM, McField M (2009) Local stressors reduce coral resilience to bleaching. PLos ONE 4: doi: 10.1371/journal.pone.0006324. PMC2708352 19623250

[22] McClanahan TR, Weil E, Maina J (2009) Strong relationship between coral bleaching and growth anomalies in massive Porites. Global Change Biology 15: 1804–1816.

[23] Pratchett MS, Wilson SK, Graham NAJ, Munday PL, Jones GP, et al. (2009) Coral bleaching and consequences for motile reef organisms: past, present and uncertain futures. In: van Oppen MJH, Lough JM, editors. Coral Bleaching: Patterns, Processes, Causes and Consequences. Berlin: Springer. pp. 139–158.

[24] Knutson DW, Buddemier RW, Smith SV (1972) Coral chronometers: seasonal growth bands in reef corals. Science 177: 270–272. 17815626 10.1126/science.177.4045.270

[25] Lough JM (2010) Climate records from corals. Wiley Interdisciplinary Reviews – Climate Change

[26] Lough JM, Cooper TF (2011) New insights from coral growth band studies in an era of rapid environmental change. Earth-Science Reviews 108: 170–184.

[27] Cooper TF, De'ath G, Fabricius KE, Lough JM (2008) Declining coral calcification in massive Porites in two nearshore regions of the northern Great Barrier Reef. Global Change Biology 14: 529–538.

[28] De'ath G, Lough JM, Fabricius KE (2009) Declining coral calcification on the Great Barrier Reef. Science 323: 116–119. 19119230 10.1126/science.1165283

[29] De'ath G, Fabricius K, Lough JM (2013) Yes-Coral calcification rates have decreased in the last twenty-five years! Marine Geology 10.1016/j.margeo.2013.09.008

[30] Cantin NE, Cohen AL, Karnauskas KB, Tarrant AM, McCorkle DC (2010) Ocean warming slows coral growth in the central Red Sea. Science 329: 322–325. 20647466 10.1126/science.1190182

[31] Tanzil JTI, Brown BE, Tudhope AW, Dunne RP (2013) Regional decline in growth rates of massive *Porites* corals in Southeast Asia. Global Change Biology 19: 3011–3023. 23744603 10.1111/gcb.12279

[32] Bak RPM, Nieuwland G, Meesters EH (2009) Coral growth rates revisited after 31 years: what is causing lower extension rates in Acropora palmata? Bulletin Marine Science 84: 287–294.

[33] Tanzil JTI, Brown BE, Tudhope AW, Dunne RP (2009) Decline in skeletal growth of the coral Porites lutea from the Andaman Sea, South Thailand between 1984 and 2005. Coral Reefs 28: 519–528.

[34] Manzello DP (2010) Coral growth with thermal stress and ocean acidification: lessons from the eastern tropical Pacific. Coral Reefs 29: 749–758.

[35] D'Olivo JP, McCulloch MT, Judd K (2013) Long-term records of coral calcification across the central Great Barrier Reef: assessing teh impacts of river runoff and climate change. Coral Reefs 10.1007/s00338-013-1071-8

[36] Cooper TF, O'Leary RA, Lough JM (2012) Growth of Western Australian Corals in the Anthropocene. Science 335: 593–596. 22301320 10.1126/science.1214570

[37] Carilli JE, Norris RD, Black BA, Walsh SM, McField M (2009) Local Stressors Reduce Coral Resilience to Bleaching. PLoS ONE 4: e6324. 19623250 10.1371/journal.pone.0006324 PMC2708352

[38] Macintyre IG, Smith SV (1974) X-radiographic studies of skeletal development in coral colonies.

[39] Budd AF, Fukami H, Smith ND, Knowlton N (2012) Taxonomic classification of the reef coral family Mussidae (Cnidaria: Anthozoa: Scleractinia). Zoological Journal of the Linnean Society 166: 465–529.

[40] Hudson EJ (1981) Growth rates in *Montastrea annularis* are a record of environmental change in Key Largo Reef Marine Sanctuary, Florida. Bulletin of Marine Science 31: 444–459.

[41] Goreau TJ, Macfarlane AH (1990) Reduced growth rates of *Montastrea annularis* following the 1987-1988 coral-bleaching event. Coral Reefs 8: 211–215.

[42] Leder JJ, Szmant AM, Swart PK (1991) The effect of prolonged “bleaching” on skeletal banding and stable isotopic composition in *Montastrea annularis* . Coral Reefs 10: 19–27.

[43] Mendes JM, Woodley JD (2002) Effect of the 1995-1996 bleaching event on polyp tissue depth, growth, reproduction and skeletal band formation in Montastraea annularis. Marine Ecology Progress Series 235: 93–102.

[44] Tudhope AW, Allison N, Tissier MDAL, Scoffin TP (1993) Growth characteristics and susceptibility to bleaching in massive Porites corals, south Thailand.

[45] Baker AC, Starger CJ, McClanahan T, Glynn PW (2004) Corals adaptive response to climate change. Nature 430: 741. 15306799 10.1038/430741a

[46] Stat M, Loh WKW, LaJeunesse TC, Hoegh-Guldberg O, Carter DA (2009) Stability of coral–endosymbiont associations during and after a thermal stress event in the southern Great Barrier Reef. Coral Reefs 28: 709–713.

[47] Sampayo EM, Ridgway T, Bongaerts P, Hoegh-Guldberg O (2008) Bleaching susceptibility and mortality of corals are determined by fine-scale differences in symbiont type. Proc Natl Acad Sci U S A 105: 10444–10449. 18645181 10.1073/pnas.0708049105 PMC2492480

[48] Carilli J, Norris RD, Black B, Walsh SM, McField M (2010) Century-scale records of coral growth rates indicate that local stressors reduce coral thermal tolerance threshold. Global Change Biology 16: 1247–1257.

[49] Lough JM, Barnes DJ (1990) Measurement of density in slices of coral skeleton: effect of densitometer beam diameter. Journal Expimental Marine Biology Ecology 143: 91–99.

[50] Lough JM, Barnes DJ (1990) Intra-annual timing of density band formation of *Porites* coral from the central Great Barrier Reef. Journal Expimental Marine Biology Ecology 135: 35–57.

[51] Chalker BE, Barnes DJ (1990) Gamma densitometry for the measurement of skeletal density. Coral Reefs 9: 11–23.

[52] Barnes DJ, Taylor RB, Lough JM (2003) Measurement of luminescence in coral skeletons. Journal Experimental Marine Biology and Ecology 295: 91–106.

[53] Lough JM, Barnes DJ, McAllister FA (2002) Luminescent lines in corals from the Great Barrier Reef provide spatial and temporal records of reefs affected by land runoff. Coral Reefs 21: 333–343.

[54] Lough JM (2007) Tropical river flow and rainfall reconstructions from coral luminescence: Great Barrier Reef, Australia. Paleoceanography 22: 10.1029/2006PA001377

[55] Hendy EJ, Gagan MK, Lough JM (2003) Chronological control of coral records using luminescent lines and evidence for non-stationary ENSO teleconnections in northeast Australia. The Holocene 13: 187–199.

[56] Lough JM, Barnes DJ (2000) Environmental controls on growth of the massive coral *Porites* . Journal Experimental Marine Biology Ecology 245: 225–243. 10.1016/s0022-0981(99)00168-9 10699212

[57] Lough JM (2011) Measured coral luminescence as a freshwater proxy: comparison with visual indices and a potential age artefact. Coral Reefs 30: 169–181.

[58] Barnes DJ, Lough JM (1992) Systematic variations in the depth of skeleton occupied by coral tissue in massive colonies of *Porites* from the Great Barrier Reef. Journal Experimental Marine Biology Ecology 159: 113–128.

[59] Barnes DJ, Lough JM, Tobin BJ (1989) Density measurements and the interpretation of X-radiographic images of slices of skeleton from the colonial hard coral *Porites* . Journal Expimental Marine Biology Ecology 131: 45–60.

[60] Barnes DJ, Lough JM (1990) Computer simulations showing the likely effects of calix architecture and other factors on retrieval of density information from coral skeletons. Journal Experimental Marine Biology Ecology 137: 141–164.

[61] Zar JH (1996) Biostatistical analysis. Englewood Cliffs, N.J.: Prentice Hall.

[62] De'ath G, Fabricius K (2010) Water quality as a regional driver of coral biodiversity and macroalgae on the Great Barrier Reef. Ecological Applications 20: 840–850. 20437968 10.1890/08-2023.1

[63] Rayner NA, Parker DE, Horton EB, Folland CK, Alexander LV, et al (2003) Global analyses of sea surface temperature, sea ice, and night marine air temperature since the late nineteenth century. Journal of Geophysical Research 108: 4407.

[64] Heron SF, Willis BL, Skirving WJ, Eakin CM, Page CA, et al (2010) Summer Hot Snaps and Winter Conditions: Modelling White Syndrome Outbreaks on Great Barrier Reef Corals. PLoS ONE 5(8): e12210. 20808912 10.1371/journal.pone.0012210 PMC2923161

[65] Liu G, Strong AE, Skirving W (2003) Remote Sensing of Sea Surface Temperatures During 2002 Barrier Reef Coral Bleaching. Eos, Transactions 84: 137–144.

[66] Lough JM (2008) Coral calcification from skeletal records revisited. Marine Ecology Progress Series 373: 257–264.

[67] Berkelmans R, Oliver J (1999) Large-scale bleaching of corals on the Great Barrier Reef. Coral Reefs 18: 55–60.

[68] Michalek-Wagner K, Willis BL (2001) Impacts of bleaching on the soft coral *Lobophytum compactum.* I. Fecundity, fertilization and offspring viability. Coral Reefs 19: 231–239.

[69] Bellwood DR, Hughes TP, Folke C, Nystrom M (2004) Confronting the coral reef crisis. Nature 429: 827–833. 15215854 10.1038/nature02691

[70] Jones AM, Berkelmans R, van Oppen MJH, Mieog JC, Sinclair W (2008) A community change in the algal endosymbionts of a scleractinian coral following a natural bleaching event: field evidence of acclimatization. Proceedings of the Royal Society B: Biological Sciences 275: 1359–1365. 10.1098/rspb.2008.0069 PMC2367621 18348962

[71] Marshall AT, Clode P (2004) Calcification rate and the effect of temperature in a zooxanthellate and an azooxanthellate scleractinian reef coral. Coral Reefs 23: 218–224.

[72] Rodrigues LG, Grottoli AG (2007) Energy reserves and metabolism as indicators of coral recovery from bleaching. Limnology and Oceanography 52: 1874–1882.

[73] Thornhill DJ, Rotjan RD, Todd BD, Chilcoat GC, R I-P, et al (2011) A connection between colony biomass and death in Caribbean reef-building corals. PLoS ONE 6: e29535. 22216307 10.1371/journal.pone.0029535 PMC3245285

[74] Pandolfi JM, Connolly SR, Marshall DJ, Cohen A (2011) Projecting coral reef futures under global warming and ocean acidification. Science 333: 418–422. 21778392 10.1126/science.1204794

[75] Thompson DM, van Woesik R (2009) Corals escape bleaching in regions that recently and historically experienced frequent thermal stress. Proceedings of the Royal Society B: Biological Sciences 276: 2893–2901. 10.1098/rspb.2009.0591 PMC2817205 19474044

[76] Donner SD, Skirving WJ, Little CM, Oppenheimer M, Hoegh-Guldberg OVE (2005) Global assessment of coral bleaching and required rates of adaptation under climate change. Global Change Biology 11: 2251–2265. 10.1111/j.1365-2486.2005.01073.x 34991281

[77] Sweatman H, Delean S, Syms C (2011) Assessing loss of coral cover on Australia's Great Barrier Reef over two decades, with implications for longer-term trends. Coral Reefs 30: 521–531.

[78] De'ath G, Fabricius K, Sweatman H, Puotinen M (2012) The 27-year decline of coral cover on the Great Barrier Reef and its causes. Proceedings of the National Academy of Sciences 109: 17995–17999. 10.1073/pnas.1208909109 PMC3497744 23027961

[79] Osborne K, Dolman AM, Burgess SC, Johns KA (2011) Disturbance and the Dynamics of Coral Cover on the Great Barrier Reef (1995–2009). PLoS ONE 6: e17516. 21423742 10.1371/journal.pone.0017516 PMC3053361

[80] Langdon C, Atkinson MJ (2005) Effect of elevated pCO2 on photosynthesis and calcification of corals and interactions with seasonal change in temperature/irradiance and nutrient enrichment. J Geophys Res 110: C09S07.

[81] Marubini F, Ferrier-Pagès C, Furla P, Allemand D (2008) Coral calcification responds to seawater acidification: a working hypothesis towards a physiological mechanism. Coral Reefs 27: 491–499.

